# Do commercial whitening dentifrices increase enamel erosive tooth wear?

**DOI:** 10.1590/1678-7757-2019-0163

**Published:** 2020-03-27

**Authors:** Ana Clara Correa Duarte SIMÕES, Aline DIONIZIO, João Victor Frazão CÂMARA, Isabela Tomazini SABINO-ARIAS, Flávia Mauad LEVY, Talita Mendes Oliveira VENTURA, Nathalia Rabelo BUZALAF, Thiago Beltrami Dias BATISTA, Ana Carolina MAGALHÃES, Sonia GROISMAN, Marília Afonso Rabelo BUZALAF

**Affiliations:** 1 Universidade Federal do Rio de Janeiro Faculdade de Odontologia Departamento de Odontologia Social e Preventiva Rio de JaneiroRio de Janeiro Brasil Universidade Federal do Rio de Janeiro, Faculdade de Odontologia, Departamento de Odontologia Social e Preventiva, Rio de Janeiro, Rio de Janeiro, Brasil.; 2 Universidade de São Paulo Faculdade de Odontologia de Bauru Departamento de Ciências Biológicas BauruSão Paulo Brasil Universidade de São Paulo, Faculdade de Odontologia de Bauru, Departamento de Ciências Biológicas, Bauru, São Paulo, Brasil.; 3 Pontifícia Universidade Católica do Paraná Departamento de Odontologia CuritibaParaná Brasil Pontifícia Universidade Católica do Paraná, Departamento de Odontologia, Curitiba, Paraná, Brasil.

**Keywords:** Tooth erosion, Toothpastes, Tooth bleaching agents, *In vitro* techniques

## Abstract

**Objective:**

This *in vitro* study evaluated the effect of commercial whitening dentifrices on erosive tooth wear (ETW) of bovine enamel samples, in comparison with commercial regular dentifrices.

**Methodology:**

Sixty bovine crowns were embedded in acrylic resin, polished and then had their baseline profile determined. They were randomly assigned to 5 groups (n=12/group), according to the type of commercial dentifrice to be tested: GI – Crest Anti-cavity Regular; GII – Crest 3D White; GIII – Colgate Total 12 Clean Mint; GIV – Colgate Optic White; GV – Placebo (negative control, fluoride-free dentifrice). The samples were submitted to daily erosive and abrasive challenges for 3 days. The erosive challenges were performed 3 times a day by immersing the specimens in 0.1% citric acid solution (pH 2.5) for 90 s. Each day after the first and last erosive challenges, the specimens were subjected to the abrasive challenge for 15 s, using a toothbrushing machine (Biopdi, São Carlos, SP, Brazil), soft toothbrushes and slurry (1:3 g/ml) of the tested toothpastes (1.5 N). The specimens were kept in artificial saliva between the challenges. The final profile was obtained and the ETW (µm) was calculated. Data were analyzed by Kruskal-Wallis and Dunn’s tests (p<0.05).

**Results:**

All dentifrices tested significantly reduced the enamel wear in comparison with the Placebo, except GIII. The median (95% CI) ETW was 1.35 (1.25-1.46)^bc^ for GI, 1.17 (1.01-1.34)^cd^ for GII, 1.36 (1.28-1.45)^ab^ for GIII, 1.08 (1.04-1.14)^d^ for GIV and 2.28 (2.18-2.39)^a^ for GV.

**Conclusion:**

When dentifrices from the same manufacturer were compared, the whitening dentifrices led to similar or less wear than the regular ones.

## Introduction

Erosive tooth wear (ETW) is the loss of dental hard tissue caused by the interplay between the exposure to nonbacterial acids and abrasive forces, and the action of these acids is its primary etiological factor.^1^ Due to its increasing prevalence^2^ and adverse consequences, ETW has been a matter of concern in the dental community in the last decades,^3,4^ and appropriate preventive measures must be implemented for high risk patients.^5^

The evidence on the efficacy of fluoridated dentifrices to prevent ETW is much less clear^6^ than it is for caries prevention.^7^ This may be related to the lack of clinical studies on the topic but also to the fact that the erosive challenge is stronger than the carious one due to the lower pH of the dietary and intrinsic acids compared with the bacterial acids.^8^ Moreover, dentifrices are used during brushing, which means that depending on how toothbrushing is performed and on type of dentifrice and toothbrush, dentifrices may either increase or decrease the wear degree.^9,10^

Considering that abrasion of eroded enamel increases with increasing abrasivity of the dentifrice,^11,12^ dentifrices with high abrasivity should not be used by patients at high risk for ETW.^9,13^ From the practical point of view, following this recommendation is very difficult for the patients, since information on the abrasivity of the dentifrices is not available on the labels of the product. The general advice is to avoid whitening dentifrices,^9^ which might have higher abrasivity in order to optimize the removal of extrinsic stains.^14^ However, information on the association between the use of whitening dentifrices and the increased ETW are contradictory. While some studies have reported higher ETW degrees when whitening dentifrices are used compared with conventional ones,^15,16^ others have not.^17,18^ Moreover, a recent study showed a distinct abrasive potential for whitening dentifrices with different technologies, such as disodium pyrophosphate, “blue light”, tetrasodium pyrophosphate and tetrapotassium pyrophosphate.^19^ These inconsistencies in the literature might occur due to the fact that whitening products act via the presence of abrasive, chemical, or optical agents, alone or in combination.^20^ For this reason, studies evaluating the effect of whitening dentifrices on ETW are necessary, since new products are launched into the market. Thus, this *in vitro* study sought to evaluate the effect of commercial whitening dentifrices on ETW of bovine enamel samples compared with regular commercial dentifrices. The null hypothesis tested was that brushing with the whitening dentifrices does not increase the ETW degree in comparison with the regular dentifrices.

## Methodology

### Preparation of enamel specimens

Sixty enamel specimens were prepared from freshly extracted bovine incisors that had been stored in 0.1% thymol solution (pH 7). The crown and root were separated using a cutting machine (Maruto, Kasuga, Tokyo, Japan) and a diamond disc (Maruto, Kasuga, Tokyo, Japan). The crowns were coupled to a prefabricated silicone mold (Biopdi, São Carlos, São Paulo, Brazil) and embedded in auto polymerizing acrylic resin with the labial surface exposed. After polymerization, the samples were polished using silicon carbide sandpapers (320, 600 and 1200 grades of Al_2_O_3_ papers; Buehler, Lake Bluff, Illinois, USA) and cleaned for 5 minutes in deionized water by sonication (Ultrasound T7 Thornton, Unique, Indaiatuba, São Paulo, Brazil), with a frequency of 40KHz. Then the baseline profile was measured using a contact profilometry. Thereafter, 2/3 of the surfaces of the specimens were protected with nail polish (Risqué, São Paulo, São Paulo, Brazil), to obtain two control areas (at the outer thirds of the specimens), leaving the central third free.

**Figure 1:**
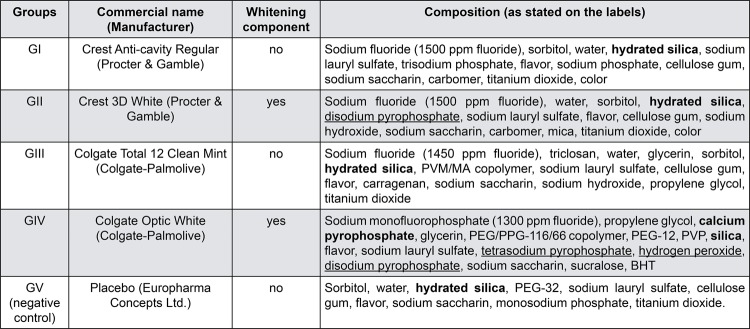
Composition of the dentifrices evaluated Abrasive components are highlighted in bold. Chemical whitening components are underlined.

The enamel specimens were randomly distributed (using Microsoft Excel) into 5 groups of 12 specimens each, according to the type of commercial dentifrice to be tested: GI – Crest Anti-cavity Regular (Procter & Gamble, Cincinnati, Ohio, USA); GII – Crest 3D White (Procter & Gamble, Cincinnati, Ohio, USA); GIII – Colgate Total 12 Clean Mint (Colgate-Palmolive, Piscataway, NJ, USA); GIV – Colgate Optic White (Colgate-Palmolive, Piscataway, NJ, USA), GV – Placebo (negative control, fluoride-free dentifrice, Europharma Concepts Ltd., Lehinch, Clare, Ireland). The composition of the dentifrices is described in Figure 1. Briefly, the dentifrices in groups I to IV are fluoridated and the dentifrice in group V is fluoride-free (negative control). In groups I and II both dentifrices were manufactured by Procter & Gamble, being I and II regular and whitening dentifrices, respectively. Similarly, dentifrices III and IV were manufactured by Colgate, being III and IV regular and whitening dentifrices, respectively. Sample size calculation was based on the study by Moron, et al.^21^ (2013). Considering the mean and SDs of placebo and 1100 ppm fluoride dentifrices, a sample size of 11 specimens would be required to obtain an α=5% and β=80%.

### Erosive-abrasive cycles and treatments

The specimens were subjected to daily erosive and abrasive challenges for 3 days. The erosive challenges were performed 3 times a day by immersing the specimens in 0.1% citric acid solution (pH 2.5, 30 mL/sample) for 90 s min at 25°C under gentle agitation.^22^ The samples were then washed with deionized water (10 s) and immersed for 2 h in artificial saliva^23^ (pH 6.8, 30 mL/sample) at 25°C for 2 h between the erosive challenges.

Each day shortly after the first and last erosive challenges, the specimens were subjected to the abrasive challenge for 15 s, using a toothbrushing machine (Biopdi, São Carlos, São Paulo, Brazil), soft toothbrushes (5460 ultrasoft Curaprox^®^, Kriens, Switzerland, 1 toothbrush/sample) and slurry of the tested toothpastes in water (1:3 g/ml) with final volume of 15 mL/sample, under standardized speed (3 linear movements/s) and force (1.5 N), at 37°C.^19,24^ The specimens were maintained in artificial saliva overnight to complete a 24 h cycle. The citric acid was renewed at each erosive challenge and the artificial saliva was daily replaced by a new one.

After 3 days, the nail varnish was removed with commercial acetone and the final profile was obtained to provide the ETW calculation.

### Contact profilometry

The ETW was measured using a contact profilometer (Mahr Perthometer, Göttingen, Germany). Five equidistant surface scans of each sample were performed (5 mm of reading, 250 μm apart, area: 5 mm^2^) at the baseline and after the experimental period. To allow repeatability, the samples had an identification mark (small drillings made with ¼ bur) and two scratches delimitating the exposed area. They were inserted into a metal device (x and y axes determination, reproducibility 0.08 µm), to allow the accurate stylus repositioning at each measurement. The baseline profile was compared with the final one using the software Marh Surf XCR20 for the enamel loss calculation. The scans were superposed, and the average depth of the under-the-curve area was calculated (μm).

### Statistical analysis

The GraphPad Instat software for Windows version 3.0 (GraphPad software Inc., La Jolla, CA, USA) was used. The data were analyzed by Kruskall-Wallis and Dunn’s test after testing the equality of variances and normal errors distribution. The significance level was set at 5%.

## Results

All tested dentifrices, except Colgate Total 12 Clean Mint, significantly reduced the enamel wear in comparison with the Placebo (Table 1). The lowest wear was found for the Colgate Optic White that performed significantly better than the other dentifrices, except for the Crest 3D White. When dentifrices from the same manufacturer were compared, the whitening dentifrices led to similar or less wear than the regular ones.

**Table 1:** Median wear of enamel (µm) after treatment with dentifrices containing whitening components or not

Dentifrice	Fluoride/Whitening	Median wear (95% CI)	Mean of Ranks
Crest Anti-cavity Regular	1500 ppm (NaF)/No	1.32 (1.25-1.46)^bc^	33.33
Crest 3D White	1500 ppm (NaF)/Yes	1.11 (1.00-1.38)^cd^	15.63
Colgate Total 12 Clean Mint	1450 ppm (NaF)/No	1.31 (1.28-1.45)^ab^	36.09
Colgate Optic White	1300 ppm (MFP)/Yes	1.08 (1.04-1.14)^d^	11.96
Placebo	No/No	2.28 (2.18-2.39)^a^	53.50

Values followed by distinct superscripts are significantly different (Kruskal-Wallis and Dunn´s test, p<0.05). n=12

## Discussion

This study was developed based on the increased concern regarding ETW^4^, the recommendation to at-risk patients on avoiding the use of whitening dentifrices due to their potentially increased abrasivity,^9,13^ and the contradictory results found in the literature regarding the abrasive potential of these dentifrices.^15-19^ According to the results of this study, the null hypothesis was accepted, since brushing with the evaluated commercial whitening dentifrices did not increase the enamel wear degree in comparison with the regular ones. In fact, the commercial whitening dentifrices led to similar or less wear when compared with the regular commercial dentifrices.

The model of this study involved an *in vitro* erosive-abrasive pH-cycling design, using bovine enamel specimens. Bovine teeth, despite less mineralized,^25^ are easier to obtain and are regarded as suitable substitutes for human teeth in studies with protocol similar to this one.^26,27^ To simulate extrinsic erosive demineralization, the specimens were immersed for 90 s in 0.1% citric acid solution (pH 2.5), following previous studies.^19,28^ Some studies, especially those conducted years ago, employ longer exposure times, such as 5^29^ or even 10 min.^30^ However, shorter periods, such as the one employed in this study, are more appropriate since they are able to demineralize the enamel without changing the degree of saturation of the solution and/or its pH value,^31^ simulating a mild erosive challenge. The erosive challenges were performed for 3 days, 3 times *per* day. In previous studies, the erosive challenges were conducted for longer periods (5-7 days),^19,28^ but in this study significant differences could be observed at 3 days, using profilometry as a response variable between the placebo dentifrice and the commercial ones. The abrasive challenge employed in this study was mild (45 linear movements during 15 s, under a 1.5 N force), similarly to other studies.^19,24^ In fact, wide variation is found in the literature regarding the number of movements and the brushing force, but reducing the duration and frequency of abrasion to better simulate the clinical condition is advised,^31^ which is in-line with this study. In fact, a previous study assessed 4 healthy volunteers’ habitual toothbrushing force and the average force (±SD) was 1.50±0.05 N.^24^ Regarding the number of movements, a recent video observation study evaluating the brushing motion patterns in adults revealed that mean brushing duration was 135 s, predominantly on vestibular surfaces, but the volunteers tended to move frequently (35 times between the sextants).^32^ This means that the number of strokes employed in this study (45 during 15 s) is within what is expected in the clinical condition, considering only one surface. Moreover, the abrasive challenges were performed twice a day to simulate the clinical condition, since most people brush their teeth twice a day. It is also important to mention that this study used an ultrasoft toothbrush (5460 Curaprox). There was no special reason for this besides the fact that similar studies have been using it.^19^ However, this might have not influenced the pattern of results, since the toothbrush filament stiffness, at least for enamel, plays only a very minor role in ETW.^10,11^

Among the active ingredients in the dentifrices evaluated, all the commercial ones had fluoride (range between 1300 and 1500 ppm), as sodium fluoride (NaF) or sodium monofluorophosphate (MFP). In fact, the effect of monovalent fluoride compounds in the dentifrices to reduce erosion and abrasion is limited, with more promising results obtained for SnF_2_-containing dentifrices.^9,33^ Interestingly, the lowest wear was found for the MFP-containing dentifrice. However, this effect was not related to MFP, since this study did not use any technique to break the covalent bond between fluoride and phosphate, which means that the amount of available fluoride was negligible.^30^ Thus, the presence of fluoride in the tested dentifrices in this study might not explain the differences found between the distinct formulations. Regarding the commercial non-whitening dentifrices, the inclusion criteria were to have dentifrices most commonly used from both manufacturers. In addition, since this study had, from the same manufacturer, one whitening and one non-whitening dentifrice, as a negative control a fluoride-free dentifrice was included, despite the role of fluoride against ETW is not as well established as that against caries.^8^ Other options for negative controls would be not to brush (erosion only)^19^ or brushing with water (to reveal the effect of toothbrush filament stiffness). However, it has been shown that the abrasivity of the dentifrice is more important than the toothbrush filament stiffness, at least for the enamel.^10,11^

Whitening dentifrices contain abrasive and whitening agents to remove extrinsic stains from the tooth surface. All the dentifrices evaluated, including placebo, had silica as abrasive agent. However, the number of abrasive agents, as well as the size, hardness and shape of the particles, are unknown since information regarding RDA/REA is not displayed on the labels. These elements are determining factors in the degree of abrasivity^34^, and not knowing this information became a limitation of this study. No linear relationship is observed between the amount of silica present in the dentifrices and the degree of enamel loss under erosive and abrasive conditions.^35^ It was recently shown that for dentifrices containing fluoride and tin, tissue loss increases up to a silica content of 10% but decreases significantly with higher amounts (20% silica is similar to the silica-free formulation).^35^

The whitening agent present in the commercial whitening dentifrices is pyrophosphate, but Colgate Optic White also contains hydrogen peroxide. While some studies report that dentifrices containing silica and pyrophosphate lead to greater enamel wear in comparison with silica-only dentifrices,^15,16,19^ the results of other studies^17,29,36^ agree with this one. The contradictory results might be explained by the distinct protocols employed in different studies. Regarding the studies that found a higher degree of wear for the dentifrices containing silica and pyrophosphate in comparison with those containing silica-only: one employed more concentrated slurry (1 part of dentifrice for 2 parts of water),^16^ the other employed longer erosion and abrasion cycles^19^ and another one was conducted *in situ* testing both sound and softened enamel.^15^ Interestingly, there was no difference between regular and whitening toothpastes for eroded enamel in the latter study, while for sound enamel the whitening dentifrice significantly increased the enamel wear.^15^ The study by Mosquim, et al.^19^ (2017) had a very similar protocol to that of this study, despite the erosive cycles, which were performed for 7 days. Interestingly, in the study by Mosquim, et al.^19^ (2017), whitening dentifrices containing silica and pyrophosphate led to higher ETW than the dentifrices containing silica-only. One could speculate that the absence of difference in the degree of wear between whitening and conventional commercial dentifrices in this study is due to the shortest period of erosive and abrasive challenges. However, in the *in situ* study by Joiner, et al.^36^ (2008) the higher degree of ETW found for whitening dentifrices in comparison with conventional ones at 4 weeks was not observed at the 12-week evaluation.

## Conclusion

In conclusion, the results of this study do not support the assumption that brushing with whitening dentifrices increases the degree of ETW in comparison with regular dentifrices. Thus, the recommendation that patients at high risk for ETW should avoid the use of whitening dentifrices lacks support in the current literature. Additional studies using methodologies that more closely resemble the clinical condition should be conducted to add evidence on this matter, considering the increasing concern regarding ETW.

## References

[1] - Shellis RP, Ganss C, Ren Y, Zero DT, Lussi A. Methodology and models in erosion research: discussion and conclusions. Caries Res. 2011;45 Suppl 1:69-77. doi: 10.1159/000325971 10.1159/000325971 21625135

[2] - Schlueter N, Luka B. Erosive tooth wear - a review on global prevalence and on its prevalence in risk groups. Br Dent J. 2018;224(5):364-70. doi: 10.1038/sj.bdj.2018.167 10.1038/sj.bdj.2018.167 29495027

[3] - Carvalho TS, Colon P, Ganss C, Huysmans MC, Lussi A, Schlueter N, et al. Consensus Report of the European Federation of Conservative Dentistry: Erosive tooth wear diagnosis and management. Swiss Dent J. 2016;126(4):342-6. 10.61872/sdj-2016-04-143 27142130

[4] - Carvalho TS, Colon P, Ganss C, Huysmans MC, Lussi A, Schlueter N, et al. Consensus report of the European Federation of Conservative Dentistry: erosive tooth wear--diagnosis and management. Clin Oral Investig. 2015;19(7):1557-61. doi: 10.1007/s00784-015-1511-7 10.1007/s00784-015-1511-7 26121968

[5] - Buzalaf MA, Magalhães AC, Rios D. Prevention of erosive tooth wear: targeting nutritional and patient-related risks factors. Br Dent J. 2018;224(5):371-8. doi: 10.1038/sj.bdj.2018.173 10.1038/sj.bdj.2018.173 29495031

[6] - Ganss C, Schulze K, Schlueter N. Toothpaste and erosion. Monogr Oral Sci. 2013;23:88-99. doi: 10.1159/000350475 10.1159/000350475 23817062

[7] - Wong MC, Clarkson J, Glenny AM, Lo EC, Marinho VC, Tsang BW, et al. Cochrane reviews on the benefits/risks of fluoride toothpastes. J Dent Res. 2011;90(5):573-9. doi: 10.1177/0022034510393346 10.1177/0022034510393346 21248357

[8] - Magalhães AC, Wiegand A, Rios D, Buzalaf MA, Lussi A. Fluoride in dental erosion. Monogr Oral Sci. 2011;22:158-70. doi: 10.1159/000325167 10.1159/000325167 21701198

[9] - Magalhães AC, Wiegand A, Buzalaf MA. Use of dentifrices to prevent erosive tooth wear: harmful or helpful? Braz Oral Res. 2014;28 Spec No:1-6. doi: 10.1590/S1806-83242013005000035 10.1590/S1806-83242013005000035 24554098

[10] 10 - Lippert F, Arrageg MA, Eckert GJ, Hara AT. Interaction between toothpaste abrasivity and toothbrush filament stiffness on the development of erosive/abrasive lesions *in vitro*. Int Dent J. 2017;67(6):344-50. doi: 10.1111/idj.12305 10.1111/idj.12305 PMC9378893 28574173

[11] 11 - Wiegand A, Schwerzmann M, Sener B, Magalhães AC, Roos M, Ziebolz D, et al. Impact of toothpaste slurry abrasivity and toothbrush filament stiffness on abrasion of eroded enamel - an *in vitro* study. Acta Odontol Scand. 2008;66(4):231-5. 10.1080/00016350802195041 18622830

[12] - Wiegand A, Kuhn M, Sener B, Roos M, Attin T. Abrasion of eroded dentin caused by toothpaste slurries of different abrasivity and toothbrushes of different filament diameter. J Dent. 2009;37(6):480-4. doi: 10.1080/00016350802195041 10.1016/j.jdent.2009.03.005 19346053

[13] 13 - Buedel S, Lippert F, Zero DT, Eckert GJ, Hara AT. Impact of dentifrice abrasivity and remineralization time on erosive tooth wear *in vitro*. Am J Dent. 2018;31(1):29-33. 29630802

[14] - Van Loveren C, Duckworth RM. Anti-calculus and whitening toothpastes. Monogr Oral Sci. 2013;23:61-74. doi: 10.1159/000350698 10.1159/000350698 23817060

[15] 15 - Turssi CP, Faraoni JJ, Rodrigues AL Jr, Serra MC. An *in situ* investigation into the abrasion of eroded dental hard tissues by a whitening dentifrice. Caries Res. 2004;38(5):473-7. 10.1159/000079629 15316192

[16] 16 - Nakamura M, Kitasako Y, Nakashima S, Sadr A, Tagami J. Impact of toothpaste on abrasion of sound and eroded enamel: an *in vitro* white light interferometer study. Am J Dent. 2015;28(5):268-72. 26714344

[17] - Joiner A, Weader E, Cox TF. The measurement of enamel wear of two toothpastes. Oral Health Prev Dent. 2004;2(4):383-8. 16296257

[18] - Joiner A, Collins LZ, Cox TF, Pickles MJ, Weader E, Liscombe C, et al. The measurement of enamel and dentine abrasion by tooth whitening products using an in situ model. Int Dent J. 2005;55(3 Suppl 1):194-6. doi: 10.1111/j.1875-595x.2005.tb00059.x 10.1111/j.1875-595x.2005.tb00059.x 16004253

[19] - Mosquim V, Martines Souza B, Foratori Junior GA, Wang L, Magalhães AC. The abrasive effect of commercial whitening toothpastes on eroded enamel. Am J Dent. 2017;30(3):142-6. 29178759

[20] - Joiner A. Whitening toothpastes: a review of the literature. J Dent. 2010;38:E17-E24. doi: 10.1016/j.jdent.2010.05.017 10.1016/j.jdent.2010.05.017 20562012

[21] 21 - Moron BM, Miyazaki SS, Ito N, Wiegand A, Vilhena F, Buzalaf MA, et al. Impact of different fluoride concentrations and pH of dentifrices on tooth erosion/abrasion *in vitro*. Aust Dent J. 2013;58(1):106-11. doi: 10.1111/adj.12016 10.1111/adj.12016 23441800

[22] 22 - Magalhães AC, Levy FM, Rizzante FA, Rios D, Buzalaf MA. Effect of NaF and TiF(4) varnish and solution on bovine dentin erosion plus abrasion *in vitro*. Acta Odontol Scand. 2012;70(2):160-4. doi: 10.3109/00016357.2011.600711 10.3109/00016357.2011.600711 21780973

[23] 23 - Klimek J, Hellwig E, Ahrens G. Fluoride taken up by plaque, by the underlying enamel and by clean enamel from three fluoride compounds *in vitro*. Caries Res.1982;16(2):156-61. doi: 10.1159/000260592 10.1159/000260592 6951638

[24] - Voronets J, Jaeggi T, Buergin W, Lussi A. Controlled toothbrush abrasion of softened human enamel. Caries Res. 2008;42(4):286-90. doi: 10.1159/000148160 10.1159/000148160 18663297

[25] 25 - Rios D, Honorio HM, Magalhaes AC, Delbem AC, Machado MA, Silva SM, et al. Effect of salivary stimulation on erosion of human and bovine enamel subjected or not to subsequent abrasion: an *in situ*/*ex vivo* study. Caries Res. 2006;40(3):218-23. doi: 10.1159/000092229 10.1159/000092229 16707870

[26] - Attin T, Wegehaupt F, Gries D, Wiegand A. The potential of deciduous and permanent bovine enamel as substitute for deciduous and permanent human enamel: erosion-abrasion experiments. J Dent. 2007;35(10):773-7. doi: 10.1016/j.jdent.2007.07.007 10.1016/j.jdent.2007.07.007 17709163

[27] - Laurance-Young P, Bozec L, Gracia L, Rees G, Lippert F, Lynch RJ, et al. A review of the structure of human and bovine dental hard tissues and their physicochemical behaviour in relation to erosive challenge and remineralisation. J Dent. 2011;39(4):266-72. doi: 10.1016/j.jdent.2011.01.008. 10.1016/j.jdent.2011.01.008 21277346

[28] - Cassiano LP, Charone S, Souza JG, Leizico LC, Pessan JP, Magalhaes AC, et al. Protective effect of whole and fat-free fluoridated milk, applied before or after acid challenge, against dental erosion. Caries Res. 2016;50(2):111-6. doi: 10.1159/000444024 10.1159/000444024 26939048

[29] - Turssi CP, Messias DC, Menezes M, Hara AT, Serra MC. Role of dentifrices on abrasion of enamel exposed to an acidic drink. Am J Dent. 2005;18(4):251-5. 16296432

[30] 30 - Kato MT, Lancia M, Sales-Peres SH, Buzalaf MA. Preventive effect of commercial desensitizing toothpastes on bovine enamel erosion *in vitro*. Caries Res. 2010;44(2):85-9. doi: 10.1159/000282668 10.1159/000282668 20145397

[31] - Wiegand A, Attin T. Design of erosion/abrasion studies - insights and rational concepts. Caries Res. 2011;45 Suppl 1:53-9. doi: 10.1159/000325946. 10.1159/000325946 21625133

[32] - Ganss C, Duran R, Winterfeld T, Schlueter N. Tooth brushing motion patterns with manual and powered toothbrushes - a randomised video observation study. Clin Oral Investig. 2018;22(2):715-20. doi: 10.1007/s00784-017-2146-7 10.1007/s00784-017-2146-7 28623465

[33] - Huysmans MC, Young A, Ganss C. The role of fluoride in erosion therapy. Monogr Oral Sci. 2014;25:230-43. doi: 10.1159/000360555 10.1159/000360555 24993271

[34] - Pickles MJ. Tooth wear. Monogr Oral Sci. 2006;19:86-104. doi: 10.1159/000090587 10.1159/000090587 16374030

[35] - Ganss C, Mollers M, Schlueter N. Do abrasives play a role in toothpaste efficacy against erosion/abrasion? Caries Res. 2017;51(1):52-7. doi: 10.1159/000452867 10.1159/000452867 27992868

[36] - Joiner A, Pickles MJ, Lynch S, Cox TF. The measurement of enamel wear by four toothpastes. Int Dent J. 2008;58(1):23-8. doi: 10.1111/j.1875-595x.2008.tb00173. 10.1111/j.1875-595x.2008.tb00173.x 18350850

